# Bicarbonate Within: A Hidden Modulator of Antibiotic Susceptibility

**DOI:** 10.3390/antibiotics14010096

**Published:** 2025-01-16

**Authors:** Selvi C. Ersoy, Warren E. Rose, Richard A. Proctor

**Affiliations:** 1 The Lundquist Institute for Biomedical Innovation at Harbor-UCLA Medical Center, Torrance, CA 90502, USA; 2 David Geffen School of Medicine, University of California Los Angeles, Los Angeles, CA 90095, USA; 3 School of Pharmacy, University of Wisconsin-Madison, Madison, WI 53705, USA; warren.rose@wisc.edu; 4 Departments of Medicine and Medical Microbiology & Immunology, University of Wisconsin School of Medicine and Public Health, Madison, WI 53705, USA; rap@wisc.edu

**Keywords:** antimicrobial susceptibility testing (AST), minimum inhibitory concentration (MIC), bicarbonate (NaHCO_3_), methicillin-resistant *Staphylococcus aureus* (MRSA), β-lactams

## Abstract

Since its standardization, clinical antimicrobial susceptibility testing (AST) has relied upon a standard medium, Mueller-Hinton Broth/Agar (MHB/A), to determine antibiotic resistance. However, this microbiologic medium bears little resemblance to the host milieu, calling into question the physiological relevance of resistance phenotypes it reveals. Recent studies investigating antimicrobial susceptibility in mammalian cell culture media, a more host-mimicking environment, demonstrate that exposure to host factors significantly alters susceptibility profiles. One such factor is bicarbonate, an abundant ion in the mammalian bloodstream/tissues. Importantly, bicarbonate sensitizes methicillin-resistant *Staphylococcus aureus* (MRSA) to early-generation β-lactams used for the treatment of methicillin-susceptible *S. aureus* (MSSA). This “NaHCO_3_-responsive” phenotype is widespread among US MRSA USA300/CC8 bloodstream and skin and soft tissue infection isolates. Translationally, β-lactam therapy has proven effective against NaHCO_3_-responsive MRSA in both ex vivo simulated endocarditis vegetation (SEV) and in vivo rabbit infective endocarditis (IE) models. Mechanistically, bicarbonate appears to influence *mecA* expression and PBP2a production/localization, as well as key elements for PBP2a functionality, including the PBP2a chaperone PrsA, components of functional membrane microdomains (FMMs), and wall teichoic acid (WTA) synthesis. The NaHCO_3_-responsive phenotype highlights the critical role of host factors in shaping antibiotic susceptibility, emphasizing the need to incorporate more physiological conditions into AST protocols.

## 1. Introduction

Antimicrobial susceptibility testing (AST) is essential for assessing antimicrobial potency, guiding treatment regimens, monitoring treatment efficacy, setting clinical susceptibility and resistance breakpoints, and identifying novel therapeutic compounds. However, a variety of flaws exist in the standard AST methodology, one of which being the choice of in vitro growth medium used for such tests. While fully recapitulating the host milieu in vitro is unattainable, ignoring the influence of any host factor in these tests will invariably result in flawed interpretations of clinical antimicrobial susceptibility.

Various recent studies have demonstrated that AST in conditions that better represent host micro-environments can provide dramatically different interpretations of antimicrobial susceptibility compared to standard AST media. What is more, bacterial strains from the same species with similar susceptibilities under standard conditions may have vastly different responses to antibiotics from one another in mammalian cell culture media that more closely mimic the host environment (“host-mimicking”). A prime example of such an intricate phenomenon is the identification of a novel phenotype, termed ‘NaHCO_3_-responsive’, wherein certain methicillin-resistant *Staphylococcus aureus* (MRSA) strains display susceptibility to β-lactams in the presence of bicarbonate, including in vivo susceptibility [1,2,3,4,5]. The existence of such a phenotype demonstrates the hidden ability of bicarbonate to modulate antimicrobial susceptibility.

Herein, we will describe the problems that arise from our standard AST methodology; attempts to improve such assays with “host-mimicking” media; and the scope, mechanisms, and impact of the newly identified ‘NaHCO_3_-responsive’ phenotype.

## 2. Antimicrobial Susceptibility Testing and Its Shortcomings

Standardized AST was first developed by the National Committee for Clinical Laboratory Standards (later termed the Clinical and Laboratory Standards Institute) in the 1960s, two decades after the introduction of the antibiotic era. To establish a standardized minimum inhibitory concentration (MIC) assay, one particular growth medium, Mueller-Hinton broth/agar (MHB/A), was selected as the standard medium to be used for such testing. Interestingly, MHB was developed decades prior as a method for isolating pathogenic strains of *Neisseria* spp. and other fastidious organisms in the laboratory, with no reference to evaluating antimicrobial susceptibilities [6,7]. While a single growth medium with broad application across bacterial species has clear appeal as a universal standard, it raises the question: What do susceptibility results from a medium so divorced from human physiology *mean* in regards to susceptibility exhibited during actual infection?

To help connect in vitro MICs with clinical outcomes, in vivo pharmacokinetic-pharmacodynamic (PK-PD) indices were developed. Such PK-PD models incorporate (i) free serum concentrations of a particular antibiotic over time (pharmacokinetics), (ii) treatment outcomes from various dosing regimens in non-clinical and clinical models, and (iii) in vitro-determined MICs [8]. However, various issues arise in regard to the use of MICs for predictive PK-PD modeling. For one, as previously stated, the MIC only determines antimicrobial potency under the very specific conditions of the MIC test [8,9]. Further, PD predictions have been found to depend greatly on the growth medium used for MIC determination [10]. This indicates that our “predictions” based on in vitro-determined MICs may have limitations in predictive value for pharmacodynamic action in patients.

Despite the shortcomings of in vitro MICs, and therefore predictive PK-PD modeling, these are the foundational elements upon which clinical breakpoints are established [11,12]. Clinical breakpoints are meant to guide physician treatment practices by correlating in vitro MICs determined for a particular organism to categories describing the likelihood of a given treatment’s success (Susceptible = likely to respond; Intermediate = response undetermined, may require increased dosing; Resistant = unlikely to respond). One of the primary metrics for defining such categories is the relationship between the pharmacological breakpoint (i.e., the concentration of a drug that can be achieved in the body over a course of time) and the in vitro MIC. Such preliminary breakpoints can then be compared to clinical outcome data and revised further if necessary. Despite this rigorous methodology, the process starts on a flawed foundation, assuming in vitro AST results mirror in vivo susceptibility.

## 3. Improving MIC Predictions of In Vivo Outcomes Using “Host-Mimicking” Media

One major element that in vitro MIC testing neglects is the influence of the host immune system on selection for or against antimicrobial susceptibility. Several studies have found that host-defense peptides (as functional cationic peptides), synergize with antimicrobials (including β-lactams) to kill pathogenic organisms in vitro and in vivo [13,14,15,16]. Interestingly, Sakoulas et al. also noted that the influence of host immune factors alters the fitness costs of certain antimicrobial resistance phenotypes [17]. As such, it would be no surprise that the incorporation of “host-mimicking” factors into AST media could have far-reaching effects on antimicrobial susceptibility profiles.

In attempts to ameliorate such discrepancies between susceptibility profiles obtained in vitro vs. in vivo, many groups have attempted to recapitulate elements of the host milieu in their AST media. Some of these attempts have included large-scale screens of antibiotics against a range of pathogens in multiple host-mimicking media [2,18,19,20]. Interestingly, these studies have found that MIC testing performed in host-mimicking media can alter the clinical breakpoint determination for a substantial proportion of both Gram-positive and Gram-negative species [2,18,19]. Further, “susceptible” and “resistant” MIC results obtained in host-mimicking media are more predictive of in vivo treatment success and failure, particularly when the host-mimicking medium recapitulates the specific infection model (e.g., LPM 5.5 for *Salmonella* infection, DMEM for bacteremia; M9Glu for lung infection) [2,18,19,20].

Other groups have primarily focused on mammalian tissue culture media, such as RPMI 1640 or DMEM, to recapitulate the host environment [21,22,23]. These media are designed to support the growth of mammalian cells in vitro, thereby supplying important nutritional requirements that likely mimic the host interstitial fluids. A primary finding of these studies has been the sensitization of the multi-drug-resistant pathogen, *Acinetobacter baumannii*, among other clinically important Gram-negative pathogens, to the macrolide azithromycin (AZM) in such media [24,25,26,27,28]. These findings are of great clinical significance as AZM is a relatively safe, broad-spectrum antibiotic, widely used in clinical practice [29]. Additionally, several of these studies have found that AZM synergizes potently with the cationic antimicrobial peptides colistin and LL-37 due to increased membrane permeability resulting in enhanced AZM cellular uptake [24,25]. Multiple studies have verified the in vivo translatability of these findings in both murine and *Galleria mellonella* models [24,25,27]. Interestingly, a screen of a large drug-repurposing library identified another drug, rifabutin, with hyperactivity against *A. baumannii* in RPMI 1640 [30,31]. Further analysis revealed that rifabutin strongly synergized with colistin in RPMI 1640 and both mono- and combination therapies were effective in vivo [31,32]. Notably, as will be discussed further in later sections, RPMI 1640 and DMEM also sensitize MRSA to several β-lactams typically effective only against methicillin-susceptible *S. aureus* (MSSA) [1,2,33,34,35].

Multiple other host-like media have been devised to mimic various infection environments, including wounds, cystic fibrosis sputum, lungs, the macrophage intracellular environment, urine, and human serum [36,37,38,39,40,41]. Similar to findings in tissue culture media, much of this research has shown that bacterial regulation and response in host-mimicking media mirrors that in vivo and is a better predictor of in vivo treatment efficacy than standard AST using bacterial growth media [37,42,43]. While antimicrobial susceptibility is generally enhanced in host-mimicking media, this is not a universal finding. *Salmonella* exhibits high-level resistance to polymyxin B and colistin in LPM 5.5, a medium representing the intracellular vacuole in which they reside during infection [2,18]. Additionally, Machado and colleagues found the development of vancomycin tolerance and resistance occurred faster in RPMI 1640 compared to MHB [44]. Notably, these findings also implicate the importance of the clinical translatability of host-mimicking media as vancomycin resistance in *S. aureus* is rarely documented in MHB despite a high treatment failure rate.

As the above studies have clearly established, AST in host-mimicking conditions will generally stimulate differences in antimicrobial susceptibility profiles for a large number of pathogens and drugs compared to standard testing media. This raises the major question of what factor(s) in such media are responsible for these changes and which are most relevant to actual infections.

## 4. Bicarbonate Is a Modulator of Antimicrobial Susceptibility

One of the earliest studies identifying bicarbonate as a key modulator of antimicrobial susceptibility in *S. aureus* and *Escherichia coli* was conducted by Dorschner et al., investigating the disparity between antimicrobial peptide (AMP) activity in vitro and in vivo [45]. These differences were initially attributed to physiologic concentrations of NaCl and the presence of host serum proteins. However, systematic investigation of elements in tissue culture medium revealed that the bicarbonate ion was the key driver of enhanced susceptibility to AMPs under these conditions [45]. Further, susceptibility to AMPs could be stimulated by bacterial pre-exposure to bicarbonate, therefore revealing a specific action of bicarbonate on the bacterium rather than the antimicrobial compound. Mechanistically, bicarbonate repressed expression of the *S. aureus* stress response regulator *sigB*, resulting in decreased cell wall thickness, likely contributing to enhanced AMP susceptibility [45]. This pivotal study revealed the key impact of bicarbonate as a specific host factor dictating antimicrobial susceptibility.

Following this, Ersoy et al. discovered that bicarbonate was the primary mediator of many changes in antimicrobial resistance observed in standard AST media vs. tissue culture media [2]. This study revealed that bicarbonate altered the susceptibility of various pathogens, including MRSA, *Streptococcus pneumoniae*, and *Salmonella* spp., to multiple classes of antibiotics. Further, AST results obtained in media containing bicarbonate were more predictive of in vivo outcomes in murine infection models. Corroborating this work, a study by Farha et al. also found that bicarbonate dictated the susceptibility of a broad group of antibiotics in both methicillin-susceptible *S. aureus* (MSSA) and *E. coli* [46]. Mechanistic investigations indicated that bicarbonate dissipated the proton motive force (PMF), thereby influencing cellular respiration and antibiotic uptake.

Following these initial findings, many studies have investigated bicarbonate’s influence on antimicrobial susceptibility across various other pathogens and antimicrobial agents. Building on the findings of Dorschner et al., another study found that bicarbonate enhanced both neutrophil killing and the LL-37 susceptibility of *Pseudomonas aeruginosa* [47]. Interestingly, a study of *S. aureus* small colony variants (SCVs) found that bicarbonate actually enhanced LL-37 resistance in these isolates [48]. Further, *sigB* and *tcaR* mediated bicarbonate-stimulated LL-37 resistance in SCVs [48], contrasting with the role of *sigB* as facilitating bicarbonate-stimulated LL-37 susceptibility in wild-type *S. aureus* [45].

Others have focused on the impact of bicarbonate on AZM susceptibility, given its clinical relevance and the wealth of evidence from tissue culture media. In one such study, bicarbonate enhanced AZM susceptibility in multiple pathogens via enhanced intracellular accumulation [49]. Further in vivo studies revealed the addition of bicarbonate to a topical AZM formulation enhanced its potency in a *P. aeruginosa* wound infection model [49]. AZM also effectively reduced bacterial burdens in a systemic MRSA infection model, presumably due to physiologic concentrations of bicarbonate in the tissues and blood [49]. Another interesting study found that while bicarbonate enhanced the activity of azithromycin against *A. baumannii*, it increased resistance to a different protein synthesis inhibitor, minocycline [50]. Despite their opposing responses to bicarbonate, AZM and minocycline demonstrated synergy in both the presence and absence of bicarbonate in vitro and in a murine *A. baumannii* pneumonia model.

**Figure 1:**
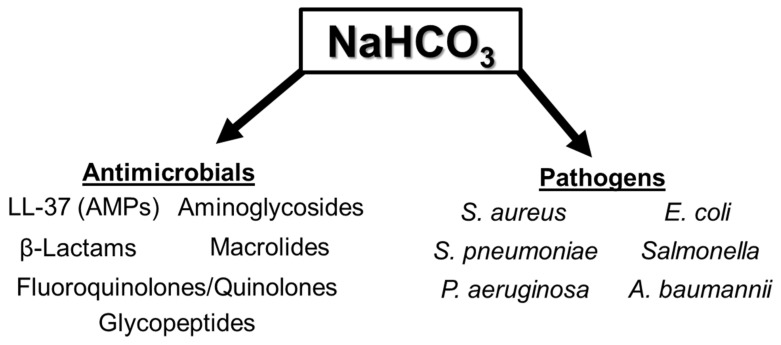
Summary of classes of antimicrobials and pathogens with bicarbonate-altered susceptibility.

Beyond its effects on LL-37 and AZM activity, bicarbonate has also been investigated for its ability to alter the effectiveness of several other antibiotic classes. Bicarbonate increased the activity of the aminoglycoside kanamycin against enteropathogenic *E. coli*, potentially due to impacts on tryptophan metabolism and iron acquisition [51,52]. Interestingly, while bicarbonate enhanced the activity of the aminoglycoside tobramycin against planktonic *P. aeruginosa*, this combination became antagonistic in biofilms, further promoting their growth [53]. Of note, however, is that bicarbonate repressed *P. aeruginosa* biofilm growth in the absence of antibiotics [54,55,56]. When tested against three different fluoroquinolones, the activity of all three drugs was reduced in tissue culture media, however, only the activity of delafloxacin was specifically affected by bicarbonate exposure alone [57]. Of particular interest is that bicarbonate has been shown to activate a peptidoglycan-degrading lysin against *Salmonella* spp. [58]. This is significant as lysins require cell wall/membrane destabilizing agents to penetrate the Gram-negative outer membrane and access the peptidoglycan layer. The role of bicarbonate as an inhibitor of cell wall synthesis will be further explored in a later section. A summary of the classes of antimicrobials and pathogens whose susceptibility is influenced by bicarbonate is provided in Figure 1.

Although many studies support the notion that the bicarbonate ion itself is a modulator of antimicrobial susceptibility, a study by Hinnu et al. aimed to rebut these findings as a consequence of changes to media pH [59]. Critically, however, the authors only investigate the impact of bicarbonate on the susceptibility of one bacteria and antibiotic combination (*S. enterica* serovar Typhimurium and AZM). Further, the authors claim that changes to AZM susceptibility were completely negated by incubation with 5% CO_2_, which maintained appropriate media pH. However, Ersoy et al. demonstrated that exposure to tissue culture media under 5% CO_2_ incubation enhanced susceptibility to AZM compared to standard AST media and removal of bicarbonate alleviated this effect in *Salmonella*, *S. aureus*, and *S. pneumoniae* [2]. Additionally, multiple studies show that the efficacy of AZM in murine models, wherein host tissue pH is tightly regulated, aligns with its predicted efficacy in bicarbonate-containing media [2,24,25,28,46]. Therefore, the body of evidence still weighs in favor of bicarbonate, rather than media pH, being a primary driver of reported changes to antimicrobial susceptibility.

Considering the many modulatory effects on antimicrobial efficacy, it is no surprise that bicarbonate has multiple physiological impacts on bacterial cells. One primary effect recognized for decades is its own direct antibacterial activity [54,60,61,62]. As previously mentioned, bicarbonate also inhibits biofilm formation via alteration of secondary intracellular messengers [54,55,56]. Additionally, bicarbonate has widespread impacts on the expression of *S. aureus* virulence factor regulators, including *sigB*, *sarA*, and *agr* [1,63,64,65]. Finally, bicarbonate depletion increases cell wall thickness, alters WTA glycosylation, and enhances resistance to cell wall lytic enzymes and detergents [66], complementing the opposite effects of bicarbonate addition on the *S. aureus* cell wall reported by Dorschner et al. [45].

## 5. Bicarbonate Sensitizes MRSA to Anti-MSSA β-Lactams

One of the major bicarbonate findings is its ability to sensitize MRSA to β-lactams used as standard-of-care therapy for MSSA strains [1,2]. This is of great potential clinical significance, considering β-lactams for susceptible *S. aureus* are relatively cheap, more effective, and less toxic than the standard anti-MRSA therapy vancomycin [67,68]. The seminal study by Ersoy et al. characterizing this phenomenon found that only certain MRSA strains were sensitized to β-lactams by bicarbonate, a phenotype termed ‘NaHCO_3_-responsive’ [1]. These strains tended to display hetero-resistant phenotypes, wherein only a small proportion or ‘sub-population’ of cells displays resistance to the antimicrobial agent, and bicarbonate suppressed resistance in these sub-populations. Additionally, bicarbonate and β-lactams synergized with LL-37 specifically in NaHCO_3_-responsive MRSA. Translationally, NaHCO_3_-responsive strains were effectively cleared by β-lactams in a rabbit model of infective endocarditis (IE) to levels comparable to the β-lactam treatment of MSSA infection. Mechanistic investigations revealed bicarbonate repressed expression of *mecA*, the key determinant of MRSA β-lactam resistance, and *sarA*, a virulence regulator associated with β-lactam resistance.

Building on these findings, several studies were undertaken to determine the overall prevalence of this phenotype among MRSA isolates from distinct infection sites and geographic locations. Interestingly, key differences were found in the frequency of NaHCO_3_ responsiveness dependent on the isolate infection source. Primarily, the NaHCO_3_-responsive phenotype was more prevalent amongst bloodstream infection (BSI) isolates than those from skin and soft tissue infections (SSTIs) [3,5]. Considering that bicarbonate levels are higher in the blood than in the skin [69], this could indicate that NaHCO_3_ responsiveness aids in bloodstream pathogenesis or dissemination from the skin to blood environs. Supporting this notion is the finding that the frequency of NaHCO_3_ responsiveness is nearly 2-fold greater in SSTI isolates that disseminated to BSI infection than in BSI isolates that originated from other sources (63% vs. 36%) [3] (Ersoy et al., *unpublished data*).

In regard to geographic distribution, the NaHCO_3_-responsive phenotype appears most frequently in USA300/CC8 strains, one of the most commonly circulating MRSA genetic backgrounds in North America [3,5,70,71]. Investigations have revealed the frequency of this phenotype is much lower in other geographic regions, such as the United Kingdom and Australia, where USA300/CC8 MRSA is less prevalent [5,72]. Such findings imply that outcomes of future clinical trials into β-lactam therapy for MRSA infections will be highly dependent on the geographic location in which the study is performed and the infection source.

Considering the clinical relevance of this phenotype, multiple approaches have been undertaken to investigate the translatability of NaHCO_3_ responsiveness in the clinic. One such study utilized an ex vivo simulated endocarditis vegetation (SEV) model to investigate the efficacy of β-lactams against NaHCO_3_-responsive vs. non-responsive MRSA [73]. The major benefit of the SEV model is the ability to mimic human PK/PD antibiotic dosing while bacteria are exposed to human components with immunologic effects (i.e., fibrin/fibrinogen and platelets) present during infective endocarditis. Further, unlike traditional in vivo models, bicarbonate levels can be regulated to directly observe the impact of specific concentrations on antibiotic PK/PD and efficacy. This study validated in vitro findings that bicarbonate stimulated enhanced β-lactam susceptibility in NaHCO_3_-responsive strains in a dose-dependent manner under ex vivo conditions [73].

Another approach to assessing the translatability of the NaHCO_3_-responsive phenotype focused on enhancing the clinical identification of MRSA strains likely to respond to β-lactam therapy [74]. Clinical microbiology laboratories utilize standard automated methodology for AST so the introduction of novel testing methods that incorporate bicarbonate would not be easily feasible. Therefore, it was important to devise a simple procedure based on currently employed methods (i.e., disk diffusion testing and whole genome sequencing) that could aid in the identification of NaHCO_3_-responsive MRSA. On this premise, an algorithm was established in which two-thirds of NaHCO_3_-responsive BSI isolates could be identified with 100% specificity based on amoxicillin-clavulanate disk diffusion testing and *mecA* and *spa* genotypes [74]. Such a formula could be easily employed by clinical microbiology labs, although further validation with a larger number of isolates from different infection sources is needed to verify its utility.

**Figure 2:**
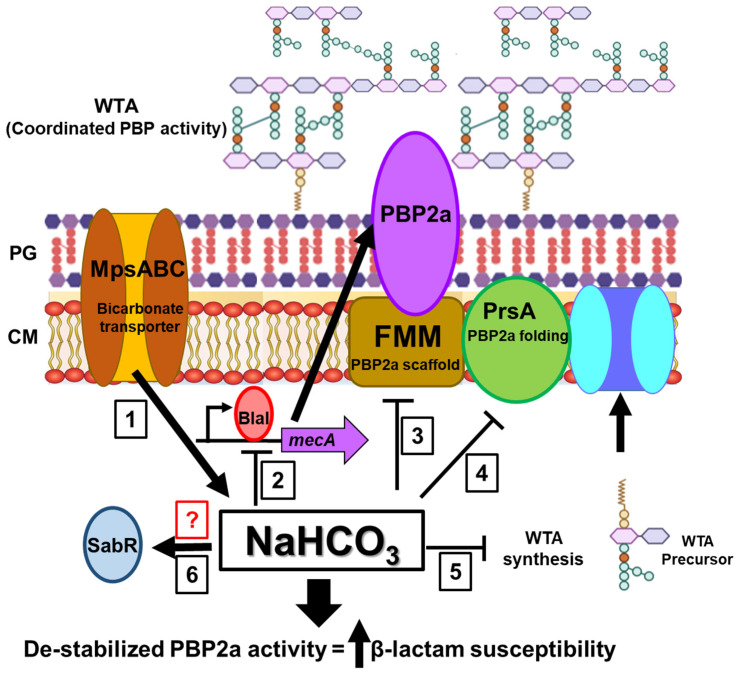
Major points of action in bicarbonate-stimulated β-lactam susceptibility in MRSA. PBP2a is the primary PBP required for peptidoglycan (PG) synthesis in the presence of β-lactam antibiotics. (1) The MpsABC bicarbonate transporter is required for intracellular accumulation of bicarbonate; deletion of *mpsABC* alleviates the NaHCO_3_-responsive phenotype. (2) Bicarbonate inhibits *mecA* transcription, likely via the *bla* regulatory system, reducing PBP2a production and localization to the cell membrane (CM). (3) Bicarbonate represses staphyloxanthin production, a key component of functional membrane microdomains (FMMs) that support PBP2a functionality. (4) Bicarbonate inhibits PrsA production and membrane localization, a chaperone that aids in proper PBP2a folding and function. (5) Bicarbonate suppresses WTA synthesis, which is necessary for coordinated PBP activity. WTA inhibition is known to sensitize MRSA to β-lactams. (6) Bicarbonate activates SabR, a potential upstream mediator of Impacts 2–5. These bicarbonate-mediated effects likely destabilize PBP2a activity, leading to increased β-lactam susceptibility.

Of course, one of the primary requirements for the eventual clinical translatability of the NaHCO_3_-responsive phenotype is establishing the molecular mechanism(s) by which bicarbonate sensitizes MRSA to β-lactams. To this end, multiple studies have been undertaken to determine key molecular and genetic factors involved in this phenotype. These are summarized in Figure 2. One of the main targets of the investigation was *mecA*, the gene that encodes the alternative penicillin-binding protein (PBP) 2a, the primary determinant of MRSA β-lactam resistance [75,76]. Consistent with this, bicarbonate suppressed the expression of *mecA* and PBP2a production and overall membrane localization [65]. Further investigations revealed that the likely mechanism of suppressed *mecA* expression was bicarbonate-mediated repression of the *bla* regulatory axis [77], the classical regulator of *mecA* expression in many clinical MRSA isolates [78,79,80]. In addition to direct impacts on *mecA*/PBP2a, bicarbonate suppressed the expression and membrane localization of the PBP2a chaperone PrsA [65], another critical component of β-lactam resistance [81,82]. Bicarbonate also suppressed staphyloxanthin production [65], regulated by *sigB*, an integral part of functional membrane microdomains necessary for PBP2a functionality [83]. Together, these data highlight the critical impacts of bicarbonate on multiple elements required for PBP2a activity.

Another recent study identified the importance of specific *mecA* genotypes in dictating susceptibility to combinations of β-lactams/β-lactamase inhibitors [84]. To evaluate the role of these specific genotypes, studies were performed in which *mecA* genotypes were “swapped” between NaHCO_3_-responsive and non-responsive strains [85]. Additional studies were performed to determine the influence of bicarbonate and specific PBP2a variants on β-lactam binding [86]. Interestingly, these studies revealed several key findings: (i) the introduction of a non-responsive *mecA* genotype into a NaHCO_3_-responsive strain eliminated bicarbonate-induced β-lactam susceptibility and PBP2a suppression; (ii) however, the introduction of a NaHCO_3_-responsive *mecA* genotype into a non-responsive strain did not generate any sensitized phenotype; and (iii) bicarbonate enhanced the ability of β-lactams to bind to both PBP2a variants but the effect was stronger in the NaHCO_3_-responsive variant. Importantly, these data indicate that while certain *mecA* genotypes/PBP2a variants may be required to maintain the NaHCO_3_-responsive phenotype, they are not sufficient to generate NaHCO_3_-responsiveness. As such, additional factors besides PBP2a are likely involved in bicarbonate-mediated β-lactam susceptibility.

Many studies have demonstrated that disruption to WTA synthesis sensitizes MRSA to β-lactams [87,88,89,90,91], indicating that WTA may be a key target of bicarbonate-mediated β-lactam sensitization. To explore this, Ersoy et al. investigated the impact of bicarbonate on WTA synthesis and associated phenotypes [92]. These studies revealed that exposure to bicarbonate inhibited WTA production in NaHCO_3_-responsive strains, resulting in several WTA-deficiency-associated phenotypes (e.g., enhanced rates of autolysis; increased frequency of aberrant cell division). Analysis of transcriptional and translational impacts of bicarbonate on key WTA synthesis genes did not reveal any direct effects on *tarO*, *tarG*, *dltA*, or *fmtA*. However, RNA-seq and qRT-PCR analyses determined that bicarbonate repressed expression of the two-epimerase genes *cap5P* and *mnaA* specifically in NaHCO_3_-responsive strains [64] (Ersoy et al., *unpublished data*). Disruption of both these functionally redundant enzymes results in the impairment of WTA synthesis [91], therefore, this represents a likely target for bicarbonate-mediated repression of WTA synthesis.

To further investigate the potential of bicarbonate as a WTA synthesis inhibitor, the effectiveness of known WTA synthesis inhibitors in combination with bicarbonate and β-lactams was evaluated [4]. This study found that WTA synthesis inhibitors were only capable of sensitizing NaHCO_3_-responsive MRSA to β-lactams and that bicarbonate further enhanced this effect. Further, WTA synthesis inhibitors strongly sensitized NaHCO_3_-responsive MRSA to the β-lactam cefuroxime in SEV and rabbit IE models. Together, these data indicate that WTA synthesis inhibitors synergize with bicarbonate to sensitize MRSA to β-lactams but this effect may be specific to NaHCO_3_-responsive strains. Larger studies with other WTA synthesis inhibitors are needed to determine the utility of such therapeutic combinations in a clinical setting.

Another possible element involved in the NaHCO_3_-responsive phenotype is the bicarbonate transporter MpsABC, required for the intracellular uptake of bicarbonate [93,94,95,96]. A study by Fan et al. observed that NaHCO_3_-responsive strains had enhanced bicarbonate uptake under ambient conditions compared to non-responsive MRSA [97]. Further, the deletion of *mpsABC* resulted in the amelioration of bicarbonate-mediated β-lactam susceptibility. These data indicate that NaHCO_3_-responsiveness may be a two-step mechanism involving, first, bicarbonate uptake and intracellular accumulation, followed by direct bicarbonate effects on the previously discussed gene expression.

In addition to targeted studies of the NaHCO_3_-responsiveness mechanism, broader RNA-seq and genome-wide association studies (GWASs) have also been undertaken [5,64]. Consistent with previous studies, RNA-seq analysis revealed that bicarbonate repressed the expression of genes within the *sigB*-*sarA*-*agr* regulatory axis, corresponding with impacts on cell-wall-anchored and stress response proteins [64,98,99]. Interestingly, GWAS analysis identified a novel gene, SAUSA300_RS00540, termed ‘*sabR*’, with distinct genotypes in NaHCO_3_-responsive and non-responsive strains [5]. Additionally, *sabR* was identified as an AraC-family transcriptional regulatory, a class of regulators known to be activated directly by bicarbonate [100,101]. Mutational studies revealed that the deletion of *sabR* eliminated the NaHCO_3_-responsive phenotype, underscoring its crucial role in the underlying mechanism [5].

Overall, these studies indicate the clinical importance and relevance of the NaHCO_3_-responsive phenotype, particularly in North America, wherein this phenotype appears to be most frequent. Translationally, in vivo studies reveal that β-lactam therapy can be efficacious against such strains and simple genotypic and phenotypic metrics could be used for their clinical identification. Broader clinical studies are required to fully adjudicate the relevance of this phenotype in human infection. Mechanistically, bicarbonate appears to influence the expression of genes within the *sigB*-*sarA*-*agr* regulon following intracellular uptake by MpsABC. The likely downstream mediators of the phenotype involve alterations in peptidoglycan and WTA synthesis, sensitizing NaHCO_3_-responsive MRSA to β-lactams. An additional regulator, *sabR*, has recently been identified but its role in the NaHCO_3_-responsive phenotype remains to be fully elucidated.

## 6. Conclusions

Recent studies have increasingly challenged the traditional use of standardized AST in MHB/A, questioning its relevance to host physiology. While we acknowledge the impact of the environment on bacterial cultivation and phenotypes, why do we overlook these factors in regard to something as crucial as antibiotic resistance? Alternative media that incorporates factors to better recapitulate the host environment, including bicarbonate, significantly impact antimicrobial susceptibility in a wide array of bacterial pathogens. Furthermore, consider the potential to discover needed antibiotic combinations and new antimicrobial compounds if the screening pipeline incorporated host-mimicking media.

Although there is strong in vitro evidence for bicarbonate as an antimicrobial agent by itself and in combination with other antibiotic compounds, clinical use of bicarbonate for infection treatment is limited. However, studies have shown promise for bicarbonate as a clinical adjuvant to control periodontal infection and improve wound dressings, as well as an alternative catheter-locking solution to prevent catheter-related bloodstream infection [61,102,103,104,105,106,107]. The use of bicarbonate in antibiotic combination therapies for more systemic infections may be challenging due to the body’s inherent buffering capacity. Further research is needed to explore strategies to overcome these challenges and better understand the potential role of bicarbonate in enhancing antibiotic efficacy in clinical settings.

While the addition of bicarbonate or other host factors into AST media has clear translational benefits, certain challenges to this approach exist. Foremost is the standardization of such media across clinical microbiology laboratories. Bicarbonate weakly buffers with atmospheric CO_2_, therefore, maintaining consistent pH utilizing such media is a challenge [59]. Such problems could be overcome through the use of additional buffers and/or CO_2_ incubators, all of which would require extensive validation. Another consideration in standardizing host-mimicking media is the variation in biological compositions, such as bicarbonate and immune factors, across different infection sites. For example, bicarbonate is abundant in the bloodstream but sparse in the skin [69,108]. This highlights the challenge of developing standardized host-mimicking media tailored to the specific conditions of different infection sites. Finally, routine clinical use of media that specifically identifies the NaHCO_3_-responsive phenotype may have less utility in regions where this phenotype appears to be infrequent (such as the UK or Australia) [5,72]. Despite these potential drawbacks to standardization for routine clinical use, the benefits of improved predictive power for antibiotic treatment efficacy may weigh in favor of host-mimicking vs. standard AST media.

Beyond AST and drug discovery, the broader incorporation of host factors, such as bicarbonate, into in vitro testing could reshape our understanding of gene function and relevant phenotypes. These factors, often overlooked or misinterpreted in the context of rich microbiologic broths, may help refine antimicrobial strategies in clinical settings. Despite the shortcomings of in vitro AST, we should consider its essential role in the overall success of antimicrobial therapy and stewardship throughout the last several decades. However, integrating host-mimicking media could refine our approaches, aligning laboratory findings more closely with clinical conditions and outcomes.

## 7. Outstanding Questions

Many outstanding questions regarding the use of host-mimicking media in AST and the NaHCO_3_-responsive phenotype still exist. In regards to AST, which host-mimicking medium would be most predictive of in vivo therapeutic outcomes? Is there one particular medium that would work broadly or would different media need to be selected based on the infection context? Other than bicarbonate, what other relevant factors are present in host-mimicking media that may dictate antimicrobial response during infection? Does the addition of such factors influence antimicrobial susceptibility in a dose-dependent fashion? Can such alternative media be practically validated for clinical translational use? In regards to the NaHCO_3_-responsive phenotype, what is the specific underlying mechanism of bicarbonate-stimulated β-lactam susceptibility? Once determined, can a simple genotypic algorithm readily identify MRSA strains likely to respond to β-lactam therapy? More broadly, can host-mimicking and/or bicarbonate-containing media improve our ability to perform in vitro phenotypic assays with greater in vivo translational relevance? Can such media be used in the drug discovery pipeline to identify novel therapeutic compounds that were previously overlooked?

## 8. Search Strategy Criteria

Data for this review were identified by searches of Google Scholar, PubMed, and references from relevant articles using the search terms “bicarbonate”, “host-mimicking media”, “establishment of MIC breakpoints”, and “MIC based PK-PD metrics”. Abstracts and reports from meetings were not included. Only articles published in English were included. There was no limitation on the publication date for inclusion.

## Data Availability

Unpublished data can be obtained by contacting selvi.ersoy@lundquist.org.

## References

[1] Ersoy S.C., Abdelhady W., Li L., Chambers H.F., Xiong Y.Q., Bayer A.S. (2019). Bicarbonate resensitization of methicillin-resistant *Staphylococcus aureus* to β-Lactam antibiotics. Antimicrob. Agents Chemother..

[2] Ersoy S.C., Heithoff D.M., Barnes L., Tripp G.K., House J.K., Marth J.D., Smith J.W., Mahan M.J. (2017). Correcting a fundamental flaw in the paradigm for antimicrobial susceptibility testing. EBioMedicine.

[3] Ersoy S.C., Otmishi M., Milan V.T., Li L., Pak Y., Mediavilla J., Chen L., Kreiswirth B., Chambers H.F., Proctor R.A. (2020). Scope and predictive genetic/phenotypic signatures of ‘bicarbonate [NaHCO_3_]-responsiveness’ and β-lactam sensitization among methicillin-resistant *Staphylococcus aureus* (MRSA). Antimicrob. Agents Chemother..

[4] Ersoy S.C., Proctor R.A., Rose W.E., Abdelhady W., Fan S.H., Madrigal S.L., Elsayed A.M., Chambers H.F., Sobral R.G., Bayer A.S. (2024). Sensitizing methicillin-resistant *Staphylococcus aureus* (MRSA) to cefuroxime: The synergic effect of bicarbonate and the wall teichoic acid inhibitor ticlopidine. Antimicrob. Agents Chemother..

[5] Ersoy S.C., Madrigal S.L., Proctor R.A., Chen L., Mediavilla J.R., Kreiswirth B.N., Flores E.A., Miller L.G., Patel R., Chambers H.F. (2024). Phenotypic and genotypic correlates of the NaHCO_3_-responsive phenotype among methicillin-resistant *Staphylococcus aureus* (MRSA) isolates from skin and soft tissue infections (SSTIs) *Clin*. Microbiol. Infect..

[6] Nizet V. (2017). The accidental orthodoxy of Drs. Mueller and Hinton. EBioMedicine.

[7] Mueller J.H., Hinton J. (1941). A protein-free medium for primary isolation of the Gonococcus and Meningococcus. Proc. Soc. Exp. Biol. Med..

[8] Landersdorfer C.B., Nation R.L. (2021). Limitations of antibiotic MIC-based PK-PD metrics: Looking back to move forward. Front. Pharmacol..

[9] Eagle H., Fleischman R., Levy M. (1953). Continuous vs. discontinuous therapy with penicillin: The effect of the interval between injections on therapeutic efficacy. N. Engl. J. Med..

[10] Berryhill B.A., Gil-Gil T., Manuel J.A., Smith A.P., Margollis E., Baquero F., Levin B.R. (2023). What’s the matter with MICs: Bacterial nutrition, limiting resources, and antibiotic pharmacodynamics. Microbiol. Spectr..

[11] MacGowan A.P., Wise R. (2001). Establishing MIC breakpoints and the interpretation of in vitro susceptibility tests. J. Antimicrob. Chemother..

[12] Humphries R.M., Abbott A.N., Hindler J.A. (2019). Understanding and addressing CLSI breakpoint revisions: A primer for clinical laboratories. J. Clin. Microbiol..

[13] Sakoulas G., Okumura C.Y., Thienphrapa W., Olson J., Nonejuie P., Dam Q., Dhand A., Pogliano J., Yeaman M.R., Hensler M.E. (2014). Nafcillin enhances innate immune-mediated killing of methicillin-resistant *Staphylococcus aureus*. J. Mol. Med..

[14] Le J., Dam Q., Schweizer M., Thienphrapa W., Nizet V., Sakoulas G. (2016). Effects of vancomycin versus nafcillin in enhancing killing of methicillin-susceptible *Staphylococcus aureus* causing bacteremia by human cathelicidin LL-37. Eur. J. Clin. Microbiol. Infect. Dis..

[15] Giacometti A., Cirioni O., Del Prete M.S., Paggi A.M., D’Errico M.M., Scalise G. (2000). Combination studies between polycationic peptides and clinically used antibiotics against Gram-positive and Gram-negative bacteria. Peptides.

[16] Li J., Fernández-Millán P., Boix E. (2020). Synergism between host defence peptides and antibiotics against bacterial infections. Curr. Top. Med. Chem..

[17] Sakoulas G., Nizet V. (2024). Measuring beta-lactam minimum inhibitory concentrations in *Staphylococcus aureus* in the clinical microbiology laboratory: Pinning the tail on the donkey. J. Clin. Microbiol..

[18] Kubicek-Sutherland J.Z., Heithoff D.M., Ersoy S.C., Shimp W.R., House J.K., Marth J.D., Smith J.W., Mahan M.J. (2015). Host-dependent induction of transient antibiotic resistance: A prelude to treatment failure. EBioMedicine.

[19] Heithoff D.M., Barnes L., Mahan S.P., Fried J.C., Fitzgibbons L.N., House J.K., Mahan M.J. (2023). Re-evaluation of FDA-approved antibiotics with increased diagnostic accuracy for assessment of antimicrobial resistance. Cell Rep. Med..

[20] Davis K.P., Morales Y., Ende R.J., Peters R., McCabe A.L., Mecsas J., Aldridge B.B. (2024). Critical role of growth medium for detecting drug interactions in Gram-negative bacteria that model in vivo responses. Mbio.

[21] Wagener V., Virtanen S. (2017). Influence of electrolyte composition (simulated body fluid vs. Dulbecco’s Modified Eagle’s medium), temperature, and solution flow on the biocorrosion behavior of commercially pure Mg. Corrosion.

[22] Moore G.E., Gerner R.E., Franklin H.A. (1967). Culture of Normal Human Leukocytes. JAMA.

[23] Dulbecco R., Freeman G. (1959). Plaque production by the polyoma virus. Virology.

[24] Lin L., Nonejuie P., Munguia J., Hollands A., Olson J., Dam Q., Kumaraswamy M., Rivera H., Corriden R., Rohde M. (2015). Azithromycin synergizes with cationic antimicrobial peptides to exert bactericidal and therapeutic activity against highly multidrug-resistant Gram-negative bacterial pathogens. EBioMedicine.

[25] Kumaraswamy M., Lin L., Olson J., Sun C.-F., Nonejuie P., Corriden R., Döhrmann S., Ali S.R., Amaro D., Rohde M. (2016). Standard susceptibility testing overlooks potent azithromycin activity and cationic peptide synergy against MDR *Stenotrophomonas maltophilia*. J. Antimicrob. Chemother..

[26] Meerwein M., Tarnutzer A., Böni M., Van Bambeke F., Hombach M., Zinkernagel A.S. (2020). Increased azithromycin susceptibility of multidrug-resistant gram-negative bacteria on RPMI-1640 agar assessed by disk diffusion testing. Antibiotics.

[27] Miller S., Goy K., She R., Spellberg B., Luna B. (2023). Antimicrobial susceptibility testing performed in RPMI 1640 reveals azithromycin efficacy against carbapenem-resistant *Acinetobacter baumannii* and predicts in vivo outcomes in galleria mellonella. Antimicrob. Agents Chemother..

[28] Lyons N., Wu W., Jin Y., Lamont I.L., Pletzer D. (2024). Using host-mimicking conditions and a murine cutaneous abscess model to identify synergistic antibiotic combinations effective against *Pseudomonas aeruginosa*. Front. Cell. Infect. Microbiol..

[29] Parnham M.J., Haber V.E., Giamarellos-Bourboulis E.J., Perletti G., Verleden G.M., Vos R. (2014). Azithromycin: Mechanisms of action and their relevance for clinical applications. Pharmacol. Ther..

[30] Luna B., Trebosc V., Lee B., Bakowski M., Ulhaq A., Yan J., Lu P., Cheng J., Nielsen T., Lim J. (2020). A nutrient-limited screen unmasks rifabutin hyperactivity for extensively drug-resistant *Acinetobacter baumannii*. Nat. Microbiol..

[31] Lee B., Yan J., Ulhaq A., Miller S., Seo W., Lu P., She R., Spellberg B., Luna B. (2021). In vitro activity of rifabutin and rifampin against antibiotic-resistant *Acinetobacter baumannii*, *Escherichia coli*, *Staphylococcus aureus*, *Pseudomonas aeruginosa*, and *Klebsiella pneumoniae*. Msphere.

[32] Cheng J., Yan J., Reyna Z., Slarve M., Lu P., Spellberg B., Luna B. (2021). Synergistic Rifabutin and Colistin Reduce Emergence of Resistance When Treating *Acinetobacter baumannii*. Antimicrob. Agents Chemother..

[33] Poudel S., Tsunemoto H., Meehan M., Szubin R., Olson C.A., Lamsa A., Seif Y., Dillon N., Vrbanac A., Sugie J. (2019). Characterization of CA-MRSA TCH1516 exposed to nafcillin in bacteriological and physiological media. Sci. Data.

[34] Rajput A., Poudel S., Tsunemoto H., Meehan M., Szubin R., Olson C.A., Lamsa A., Seif Y., Dillon N., Vrbanac A. (2019). Profiling the effect of nafcillin on HA-MRSA D712 using bacteriological and physiological media. Sci. Data.

[35] Seif Y., Poudel S., Tsunemoto H., Szubin R., Meehan M.J., Olson C.A., Rajput A., Alarcon G., Lamsa A., Dillon N. (2020). Profiling the effect of nafcillin on HA-MRSA D592 using bacteriological and physiological media. bioRxiv.

[36] Asempa T.E., Bajor H., Mullins J.H., Hartnett J., Nicolau D.P. (2021). Evaluation of Metallo-β-lactamase susceptibility testing in a physiologic medium. Microbiol. Spectr..

[37] Garcia Maset Rn Hapeshi A., Hall S., Dalgliesh R.M., Harrison F., Perrier S. (2022). Evaluation of the antimicrobial activity in host-mimicking media and in vivo toxicity of antimicrobial polymers as functional mimics of AMPs. ACS Appl. Mater. Interfaces.

[38] Sweeney E., Sabnis A., Edwards A.M., Harrison F. (2020). Effect of host-mimicking medium and biofilm growth on the ability of colistin to kill *Pseudomonas aeruginosa*. Microbiology.

[39] Thulin E., Thulin M., Andersson D.I. (2017). Reversion of high-level mecillinam resistance to susceptibility in *Escherichia coli* during growth in urine. EBioMedicine.

[40] Crabbé A., Ostyn L., Staelens S., Rigauts C., Risseeuw M., Dhaenens M., Daled S., Van Acker H., Deforce D., Van Calenbergh S. (2019). Host metabolites stimulate the bacterial proton motive force to enhance the activity of aminoglycoside antibiotics. PLoS Pathog..

[41] Ellis M.J., Tsai C.N., Johnson J.W., French S., Elhenawy W., Porwollik S., Andrews-Polymenis H., McClelland M., Magolan J., Coombes B.K. (2019). A macrophage-based screen identifies antibacterial compounds selective for intracellular Salmonella Typhimurium. Nat. Commun..

[42] Asempa T.E., Abdelraouf K., Nicolau D.P. (2020). Metallo-β-lactamase resistance in Enterobacteriaceae is an artefact of currently utilized antimicrobial susceptibility testing methods. J. Antimicrob. Chemother..

[43] Weber B.S., De Jong A.M., Guo A.B., Dharavath S., French S., Fiebig-Comyn A.A., Coombes B.K., Magolan J., Brown E.D. (2020). Genetic and chemical screening in human blood serum reveals unique antibacterial targets and compounds against *Klebsiella pneumoniae*. Cell Rep..

[44] Machado H., Seif Y., Sakoulas G., Olson C.A., Hefner Y., Anand A., Jones Y.Z., Szubin R., Palsson B.O., Nizet V. (2021). Environmental conditions dictate differential evolution of vancomycin resistance in *Staphylococcus aureus*. Commun. Biol..

[45] Dorschner R.A., Lopez-Garcia B., Peschel A., Kraus D., Morikawa K., Nizet V., Gallo R.L. (2006). The mammalian ionic environment dictates microbial susceptibility to antimicrobial defense peptides. FASEB J..

[46] Farha M.A., French S., Stokes J.M., Brown E.D. (2017). Bicarbonate alters bacterial susceptibility to antibiotics by targeting the proton motive force. ACS Infect. Dis..

[47] Siew R., Ou T.-L., Dahesh S., Akong K., Nizet V. (2022). Bicarbonate Effects on Antibacterial Immunity and Mucus Glycobiology in the Cystic Fibrosis Lung: A Review with Selected Experimental Observations. Infect. Microbes Dis..

[48] Zhang P., Wright J.A., Tymon A., Nair S.P. (2018). Bicarbonate induces high-level resistance to the human antimicrobial peptide LL-37 in *Staphylococcus aureus* small colony variants. J. Antimicrob. Chemother..

[49] Farha M.A., MacNair C.R., Carfrae L.A., El Zahed S.S., Ellis M.J., Tran H.-K.R., McArthur A.G., Brown E.D. (2020). Overcoming acquired and native macrolide resistance with bicarbonate. ACS Infect. Dis..

[50] Dillon N., Holland M., Tsunemoto H., Hancock B., Cornax I., Pogliano J., Sakoulas G., Nizet V. (2019). Surprising synergy of dual translation inhibition vs. Acinetobacter baumannii and other multidrug-resistant bacterial pathogens. EBioMedicine.

[51] Gutiérrez-Huante M., Martínez H., Bustamante V.H., Puente J.L., Sánchez J. (2015). Bicarbonate enhances the in vitro antibiotic activity of kanamycin in *Escherichia coli*. Lett. Appl. Microbiol..

[52] Gutierrez-Huante M., Martinez-Duncker M., Sauceda E., Sánchez J. (2019). The antibiotics potentiator bicarbonate causes upregulation of tryptophanase and iron acquisition proteins in *Escherichia coli*. Lett. Appl. Microbiol..

[53] Kaushik K.S., Stolhandske J., Shindell O., Smyth H.D., Gordon V.D. (2016). Tobramycin and bicarbonate synergise to kill planktonic *Pseudomonas aeruginosa*, but antagonise to promote biofilm survival. npj Biofilms Microbiomes.

[54] Jaikumpun P., Ruksakiet K., Stercz B., Pállinger É., Steward M., Lohinai Z., Dobay O., Zsembery Á. (2020). Antibacterial effects of bicarbonate in media modified to mimic cystic fibrosis sputum. Int. J. Mol. Sci..

[55] Dobay O., Laub K., Stercz B., Kéri A., Balázs B., Tóthpál A., Kardos S., Jaikumpun P., Ruksakiet K., Quinton P.M. (2018). Bicarbonate inhibits bacterial growth and biofilm formation of prevalent cystic fibrosis pathogens. Front. Microbiol..

[56] Ruksakiet K., Stercz B., Tóth G., Jaikumpun P., Gróf I., Tengölics R., Lohinai Z.M., Horváth P., Deli M.A., Steward M.C. (2021). Bicarbonate evokes reciprocal changes in intracellular cyclic di-gmp and cyclic amp levels in *Pseudomonas aeruginosa*. Biology.

[57] Holland M., Bjanes E., Nizet V., Dillon N. (2022). Bicarbonate modulates delafloxacin activity against MDR *Staphylococcus aureus* and *Pseudomonas aeruginosa*. J. Antimicrob. Chemother..

[58] Abhisingha M., Dumnil J., Pitaksutheepong C. (2023). Effect of lysin EN4 in combination with sodium bicarbonate on reduction of Salmonella in chilled and thawed chicken meat. Int. J. Food Microbiol..

[59] Hinnu M., Putrinš M., Kogermann K., Bumann D., Tenson T. (2022). Making antimicrobial susceptibility testing more physiologically relevant with bicarbonate?. Antimicrob. Agents Chemother..

[60] Corral L.G., Post L.S., Montville T.J. (1988). Antimicrobial activity of sodium bicarbonate: A research note. J. Food Sci..

[61] Li Y., Li H., Yu Z., Liu J., Lin Y., Xu J., Zhang C., Chen Q., Han X., Peng Q. (2024). Drug-free and multifunctional sodium bicarbonate/hyaluronic acid hybrid dressing for synergistic healing of infected wounds. Int. J. Biol. Macromol..

[62] Kesici S., Demirci M., Kesici U. (2019). Bicarbonate may alters bacterial susceptibility to antibiotics by targeting *Pseudomonas aeruginosa*, *Escherichia coli* and *Staphylococcus aureus*. J. Contemp. Med..

[63] Saleh M.M., Yousef N., Shafik S.M., Abbas H.A. (2022). Attenuating the virulence of the superbug *Staphylococcus aureus* by sodium bicarbonate, dexamethasone, and ascorbic acid. BMC Microbiol..

[64] Ersoy S.C., Hanson B.M., Proctor R.A., Arias C.A., Tran T.T., Chambers H.F., Bayer A.S. (2021). Impact of bicarbonate-β-lactam exposures on methicillin-resistant *Staphylococcus aureus* (MRSA) gene expression in bicarbonate-β-lactam-responsive vs. non-responsive strains. Genes.

[65] Ersoy S.C., Chambers H.F., Proctor R.A., Rosato A.E., Mishra N.N., Xiong Y.Q., Bayer A.S. (2021). Impact of Bicarbonate on PBP2a Production, Maturation, and Functionality in Methicillin-Resistant *Staphylococcus aureus* (MRSA). Antimicrob. Agents Chemother..

[66] Liberini E., Fan S.-H., Bayer A.S., Beck C., Biboy J., François P., Gray J., Hipp K., Koch I., Peschel A. (2024). *Staphylococcus aureus* stress response to bicarbonate depletion. Int. J. Mol. Sci..

[67] Rayner C., Munckhof W. (2005). Antibiotics currently used in the treatment of infections caused by *Staphylococcus aureus*. Intern. Med. J..

[68] Purrello S., Garau J., Giamarellos E., Mazzei T., Pea F., Soriano A., Stefani S. (2016). Methicillin-resistant *Staphylococcus aureus* infections: A review of the currently available treatment options. JGAR.

[69] Proksch E. (2018). pH in nature, humans and skin. J. Dermatol..

[70] Jenkins T.C., McCollister B.D., Sharma R., McFann K.K., Madinger N.E., Barron M., Bessesen M., Price C.S., Burman W.J. (2009). Epidemiology of healthcare-associated bloodstream infection caused by USA300 strains of methicillin-resistant *Staphylococcus aureus* in 3 affiliated hospitals. Infect. Control Hosp. Epidemiol..

[71] Seybold U., Kourbatova E.V., Johnson J.G., Halvosa S.J., Wang Y.F., King M.D., Ray S.M., Blumberg H.M. (2006). Emergence of community-associated methicillin-resistant *Staphylococcus aureus* USA300 genotype as a major cause of health care—Associated blood stream infections. Clin. Infect. Dis..

[72] Petersiel N., Giulieri S., Daniel D.S., Fan S.H., Ersoy S.C., Davis J.S., Bayer A.S., Howden B.P., Tong S.Y.C. (2024). Genomic investigation and clinical correlates of the in vitro β-lactam: NaHCO_3_ responsiveness phenotype among methicillin-resistant *Staphylococcus aureus* isolates from a randomized clinical trial. Antimicrob. Agents Chemother..

[73] Rose W.E., Bienvenida A.M., Xiong Y.Q., Chambers H.F., Bayer A.S., Ersoy S.C. (2019). Ability of bicarbonate supplementation to sensitize selected methicillin-resistant *Staphylococcus aureus* (MRSA) strains to β-lactam antibiotics in an ex vivo simulated endocardial vegetation model. Antimicrob. Agents Chemother..

[74] Ersoy S.C., Rose W.E., Patel R., Proctor R.A., Chambers H.F., Harrison E.M., Pak Y., Bayer A.S. (2021). A combined phenotypic-genotypic predictive algorithm for in vitro detection of bicarbonate: β-lactam sensitization among methicillin-resistant *Staphylococcus aureus* (MRSA). Antibiotics.

[75] Hartman B.J., Tomasz A. (1984). Low-affinity penicillin-binding protein associated with beta-lactam resistance in *Staphylococcus aureus*. J. Bacteriol..

[76] Chambers H.F., Sachdeva M., Kennedy S. (1990). Binding affinity for penicillin-binding protein 2a correlates with in vivo activity of β-Lactan antibiotics against methicillin-resistant *Staphylococcus aureus*. J. Infect. Dis..

[77] Ersoy S.C., Madrigal S.L., Proctor R.A., Chambers H.F., Xiong Y.Q., Bayer A.S. (2024). NaHCO_3_ Modulates the *bla* Operon and β-Lactam Susceptibility in Borderline Oxacillin-Resistant *Staphylococcus aureus* (BORSA). J. Antimicrob. Chemother..

[78] Hackbarth C.J., Chambers H.F. (1993). *blaI* and *blaR1* regulate beta-lactamase and PBP 2a production in methicillin-resistant *Staphylococcus aureus*. Antimicrob. Agents Chemother..

[79] Gregory P., Lewis R., Curnock S., Dyke K. (1997). Studies of the repressor (BlaI) of β-lactamase synthesis in *Staphylococcus aureus*. Mol. Microbiol..

[80] Elements I.W.G.o.t.C.o.S.C.C. (2009). Classification of staphylococcal cassette chromosome mec (SCCmec): Guidelines for reporting novel SCCmec elements. Antimicrob. Agents Chemother..

[81] Jousselin A., Manzano C., Biette A., Reed P., Pinho M., Rosato A., Kelley W.L., Renzoni A. (2015). The *Staphylococcus aureus* chaperone PrsA is a new auxiliary factor of oxacillin resistance affecting penicillin-binding protein 2A. Antimicrob. Agents Chemother..

[82] Jousselin A., Renzoni A., Andrey D.O., Monod A., Lew D.P., Kelley W.L. (2012). The post-translocational chaperone lipoprotein PrsA is involved in both glycopeptide and oxacillin resistance in *Staphylococcus aureus*. Antimicrob. Agents Chemother..

[83] García-Fernández E., Koch G., Wagner R.M., Fekete A., Stengel S.T., Schneider J., Mielich-Süss B., Geibel S., Markert S.M., Stigloher C. (2017). Membrane microdomain disassembly inhibits MRSA antibiotic resistance. Cell.

[84] Harrison E.M., Ba X., Coll F., Blane B., Restif O., Carvell H., Köser C.U., Jamrozy D., Reuter S., Lovering A. (2019). Genomic identification of cryptic susceptibility to penicillins and β-lactamase inhibitors in methicillin-resistant *Staphylococcus aureus*. Nat. Microbiol..

[85] Ersoy S.C., Manna A.C., Proctor R.A., Chambers H.F., Harrison E.M., Bayer A.S., Cheung A. (2022). The NaHCO_3_-responsive phenotype in methicillin-resistant *Staphylococcus aureus* (MRSA) is influenced by *mecA* genotype. Antimicrob. Agents Chemother..

[86] Ersoy S.C., Chan L.C., Yeaman M.R., Chambers H.F., Proctor R.A., Ludwig K.C., Schneider T., Manna A.C., Cheung A., Bayer A.S. (2022). Impacts of NaHCO_3_ on β-lactam binding to PBP2a protein variants associated with the NaHCO_3_-responsive versus NaHCO_3_-non-responsive phenotypes. Antibiotics.

[87] Farha M.A., Leung A., Sewell E.W., D’Elia M.A., Allison S.E., Ejim L., Pereira P.M., Pinho M.G., Wright G.D., Brown E.D. (2013). Inhibition of WTA synthesis blocks the cooperative action of PBPs and sensitizes MRSA to β-lactams. ACS Chem. Biol..

[88] Lee S.H., Wang H., Labroli M., Koseoglu S., Zuck P., Mayhood T., Gill C., Mann P., Sher X., Ha S. (2016). TarO-specific inhibitors of wall teichoic acid biosynthesis restore β-lactam efficacy against methicillin-resistant staphylococci. Sci. Transl. Med..

[89] Campbell J., Singh A.K., Santa Maria Jr J.P., Kim Y., Brown S., Swoboda J.G., Mylonakis E., Wilkinson B.J., Walker S. (2010). Synthetic lethal compound combinations reveal a fundamental connection between wall teichoic acid and peptidoglycan biosyntheses in *Staphylococcus aureus*. ACS Chem. Biol..

[90] Brown S., Xia G., Luhachack L.G., Campbell J., Meredith T.C., Chen C., Winstel V., Gekeler C., Irazoqui J.E., Peschel A. (2012). Methicillin resistance in *Staphylococcus aureus* requires glycosylated wall teichoic acids. Proc. Natl. Acad. Sci. USA.

[91] Mann P.A., Müller A., Wolff K.A., Fischmann T., Wang H., Reed P., Hou Y., Li W., Müller C.E., Xiao J. (2016). Chemical genetic analysis and functional characterization of staphylococcal wall teichoic acid 2-epimerases reveals unconventional antibiotic drug targets. PLoS Pathog..

[92] Ersoy S.C., Goncalves B., Cavaco G., Manna A.C., Sobral R.G., Nast C.C., Proctor R.A., Chambers H.F., Cheung A., Bayer A.S. (2022). Influence of sodium bicarbonate on wall teichoic acid synthesis and beta-lactam sensitization in NaHCO_3_-responsive and nonresponsive methicillin-resistant *Staphylococcus aureus*. Microbiol. Spectr..

[93] Fan S.-H., Ebner P., Reichert S., Hertlein T., Zabel S., Lankapalli A.K., Nieselt K., Ohlsen K., Götz F. (2019). MpsAB is important for *Staphylococcus aureus* virulence and growth at atmospheric CO_2_ levels. Nat. Commun..

[94] Mayer S., Steffen W., Steuber J., Götz F. (2015). The *Staphylococcus aureus* NuoL-like protein MpsA contributes to the generation of membrane potential. J. Bacteriol..

[95] Fan S.-H., Matsuo M., Huang L., Tribelli P.M., Götz F. (2021). The MpsAB bicarbonate transporter is superior to carbonic anhydrase in biofilm-forming bacteria with limited CO_2_ diffusion. Microbiol. Spectr..

[96] Fan S.-H., Liberini E., Götz F. (2021). *Staphylococcus aureus* genomes harbor only MpsAB-like bicarbonate transporter but not carbonic anhydrase as dissolved inorganic carbon supply system. Microbiol. Spectr..

[97] Fan S.H., Proctor R.A., Ersoy S.C., Manna A.C., Cheung A.L., Götz F., Chambers H.F., Bayer A.S. (2023). Role of the NaHCO_3_ transporter MpsABC in the NaHCO_3_-β-lactam-responsive phenotype in methicillin-resistant *Staphylococcus aureus*. Microbiol. Spectr..

[98] Novick R.P. (2003). Autoinduction and signal transduction in the regulation of staphylococcal virulence. Mol. Microbiol..

[99] Cheung A.L., Zhang G. (2002). Global regulation of virulence determinants in *Staphylococcus aureus* by the SarA protein family. Front. Biosci..

[100] Ibberson C.B., Whiteley M. (2019). The *Staphylococcus aureus* transcriptome during cystic fibrosis lung infection. mBio.

[101] Yang J., Tauschek M., Robins-Browne R.M. (2011). Control of bacterial virulence by AraC-like regulators that respond to chemical signals. Trends Microbiol..

[102] Taschieri S., Tumedei M., Francetti L., Corbella S., Del Fabbro M. (2022). Efficacy of 67% sodium bicarbonate toothpaste for plaque and gingivitis control: A systematic review and meta-analysis. J. Evid.-Based Dent. Pract..

[103] Bosma M.-L., Milleman K.R., Akwagyiram I., Targett D., Milleman J.L. (2018). A randomised controlled trial to evaluate the plaque removal efficacy of sodium bicarbonate dentifrices in a single brushing clinical model. BDJ Open.

[104] Parkinson C.R., Butler A., Ling M.R. (2023). Antigingivitis efficacy of a sodium bicarbonate toothpaste: Pooled analysis. Int. J. Dent. Hyg..

[105] Axe A., Patel N., Qaqish J., Ling M.R., Araga M., Parkinson C., Goyal C.R. (2024). Efficacy of an experimental toothpaste containing sodium bicarbonate, sodium hyaluronate and sodium fluoride on gingivitis. BMC Oral. Health.

[106] El-Hennawy A.S., Frolova E., Romney W.A. (2019). Sodium bicarbonate catheter lock solution reduces hemodialysis catheter loss due to catheter-related thrombosis and blood stream infection: An open-label clinical trial. Nephrol. Dial. Transplant..

[107] Ali S.S., Swami R., Shakir A., Mehta K. (2023). Safety and Efficacy of Acute Central Venous Catheters for Hemodialysis with Sodium Bicarbonate versus an Antibiotic Catheter Locking Solution. Saudi J. Kidney Dis. Transplant..

[108] Nguyen A.V., Soulika A.M. (2019). The dynamics of the skin’s immune system. Int. J. Mol. Sci..

